# Optical mapping of the dominant frequency of brain signal oscillations in motor systems

**DOI:** 10.1038/s41598-017-15046-9

**Published:** 2017-11-07

**Authors:** Feng-Mei Lu, Yi-Feng Wang, Juan Zhang, Hua-Fu Chen, Zhen Yuan

**Affiliations:** 1 Bioimaging Core, Faculty of Health Sciences, University of Macau, Macau, SAR China; 2 Key Laboratory for NeuroInformation of Ministry of Education, School of Life Science and Technology, University of Electronic Science and Technology of China, Chengdu, 610054 China; 3 Faculty of Education, University of Macau, Macau, SAR China

## Abstract

Recent neuroimaging studies revealed that the dominant frequency of neural oscillations is brain-region-specific and can vary with frequency-specific reorganization of brain networks during cognition. In this study, we examined the dominant frequency in low-frequency neural oscillations represented by oxygenated hemoglobin measurements after the hemodynamic response function (HRF) deconvolution. Twenty-nine healthy college subjects were recruited to perform a serial finger tapping task at the frequency of 0.2 Hz. Functional near-infrared spectroscopy (fNIRS) was applied to record the hemodynamic signals over the primary motor cortex, supplementary motor area (SMA), premotor cortex, and prefrontal area. We then explored the low frequency steady-state brain response (lfSSBR), which was evoked in the motor systems at the fundamental frequency (0.2 Hz) and its harmonics (0.4, 0.6, and 0.8 Hz). In particular, after HRF deconvolution, the lfSSBR at the frequency of 0.4 Hz in the SMA was identified as the dominant frequency. Interestingly, the domain frequency exhibited the correlation with behavior data such as reaction time, indicating that the physiological implication of lfSSBR is related to the brain anatomy, stimulus frequency and cognition. More importantly, the HRF deconvolution showed its capability for recovering signals probably reflecting neural-level events and revealing the physiological meaning of lfSSBR.

## Introduction

Neural oscillations at particular frequencies are essential for the investigation of various cognition functions. More importantly, the frequency of neural oscillations can be altered by nearby neurons and inter-regional information transmissions^1^. Consequently, the intrinsic, brain stimulation-induced, and cognitive-based neural oscillations can arise in different frequency bands^1–3^. Interestingly, a recent upsurge of studies on brain oscillations also reveal that the dominant frequency of neural oscillations is regional specific, constrained by anatomy, spatial distance, and cognitive hierarchy^4–8^. In particular, the dominant frequency is able to change with frequency-specific reorganization of the network’s topology during cognition^9,10^. Although the natural frequencies have been detected by using direct cortical stimulation^3^ and at resting state^2,11^, the dominant frequency in cognition can provide a fully new framework for a better understanding of brain functions.

More importantly, the execution of complex brain cognitions can last from hundreds of milliseconds to several seconds, involving both the high-frequency (>0.5 Hz) and low-frequency neural oscillations^12^. A substantial body of studies have demonstrated the roles of delta (1~4 Hz)^13^, theta (4~8 Hz)^14^, alpha (8~13 Hz)^15,16^, beta (13~30 Hz)^17^, gamma rhythms (30~150 Hz)^18^, and cross-frequency coupling activities^19,20^ by using electroencephalography (EEG) or magnetoencephalography (MEG) recordings. However, the EEG/MEG techniques are restricted to a high signal bandwidth that is generally larger than 0.5 Hz, which makes it very challenging to identify the low-frequency brain oscillation activity. As such, the neural oscillations mechanism within the low-frequency ranges (<0.5 Hz) remains unclear largely due to the deficiency of available approaches to inspect them. By contrast, fMRI studies were performed to evoke neural oscillations with cognitive tasks, which was called low frequency steady-state brain response (lfSSBR)^21–23^. lfSSBR is evoked by cognitive tasks rather than external electromagnetic stimuli. It is independent of the neurovascular coupling, which, to a large degree, can reflect neural-level activities^22,23^. Moreover, compared with steady-state evoked potentials (SSEP) revealed by EEG, lfSSBR can be induced by complex higher-order cognition^22,24^ which is able to exhibit strong responses in brain regions including the insula, fronto-parietal region, and fusiform face area in the infra-slow frequency band of 0~0.5 Hz.

Similar to fMRI, functional near-infrared spectroscopy (fNIRS) can also measure the cerebral hemodynamic responses including the concentration changes both in oxygenated hemoglobin (HbO) and deoxygenated hemoglobin (HbR) within the low-frequency ranges^25–34^. fNIRS has shown its promise to examine the low-frequency HbR and HbO oscillations underlying different stimuli tasks^27^. However, it is recognized that the hemoglobin responses measured by fNIRS are regulated by the neurovascular coupling mechanism rather than the neural activity itself. Consequently, since the traditional general linear model (GLM) heavily depends on the neurovascular coupling, the concern on whether the low-frequency HbR and HbO fluctuations really reflect the underlying processes of neural oscillations is still under debate. More importantly, it is also unknown whether the low-frequency HbO/HbR fluctuations can manifest the dominant frequency of neural oscillations. As such, it is hypothesized in this study that lfSSBR in the slow frequency bands (0.1~0.8 Hz) can be evoked by fNIRS recordings, which is able to modulate the neural oscillations in low frequency. Meanwhile, we also hypothesize that the dominant frequency of neural oscillations can be identified by fNIRS.

To test our hypothesis, 29 healthy subjects were recruited from the University of Macau campus to participate a serial finger tapping (SFT) task at a fixed frequency of 0.2 Hz. Then the power analysis was performed to extract lfSSBR based on HbO or HbR signals, in which the SSEP-like waveforms were generated across the task-related brain motor regions. The blind HRF deconvolution approach, which is established on the idea that HbO/HbR spikes are derived from the point events with non-random patterns, was adopted to generate the neural level signals from the extracted HbO/HbR measures^35,36^. By matching HbO/HbR signals with canonical HRF and its time derivative, the blind HRF deconvolution is able to eliminate the effect of hemodynamic responses in a maximal extent. As such, we can compare the lfSSBR before HRF deconvolution with that after HRF deconvolution to quantify whether the lfSSBR is cognitive task-related, which can reflect the neural oscillations. If lfSSBR does not exhibit significant difference between the cases before and after HRF deconvolution, we can claim that lfSSBR is independent of the neurovascular coupling. More specifically, we will also examine whether the dominant frequency of neural oscillations is independent of the neurovascular coupling.

## Results

### Behavioral data results

The analysis of behavioral data was performed, in which the mean and standard deviation (SD) of mean reaction time (RT) were calculated across 66 trials for each subject. The mean RT and accuracy (ACC) rates for the whole group were provide in Table 1. Extreme data (1000 ms <RT or RT <200 ms, and ±3 SD away from the participant’s own condition mean) were excluded from this study for further analysis.

**Table 1:** The participants’ mean RT and ACC for the performance of the SFT task.

Behavior results	Values
mean RT (mean ± SD)	556.50 ± 133.01
mean RT range at group level	354.72~857.86
SD RT (mean ± SD)	98.60 ± 58.93
SD RT range at group level	31.82~260.20
ACC (mean ± SD)	97% ± 4%
ACC range	83% to 100%

RT, reaction time; SD, standard deviation; ACC, accuracy.

### The effect of neurovascular coupling

**Figure 1:**
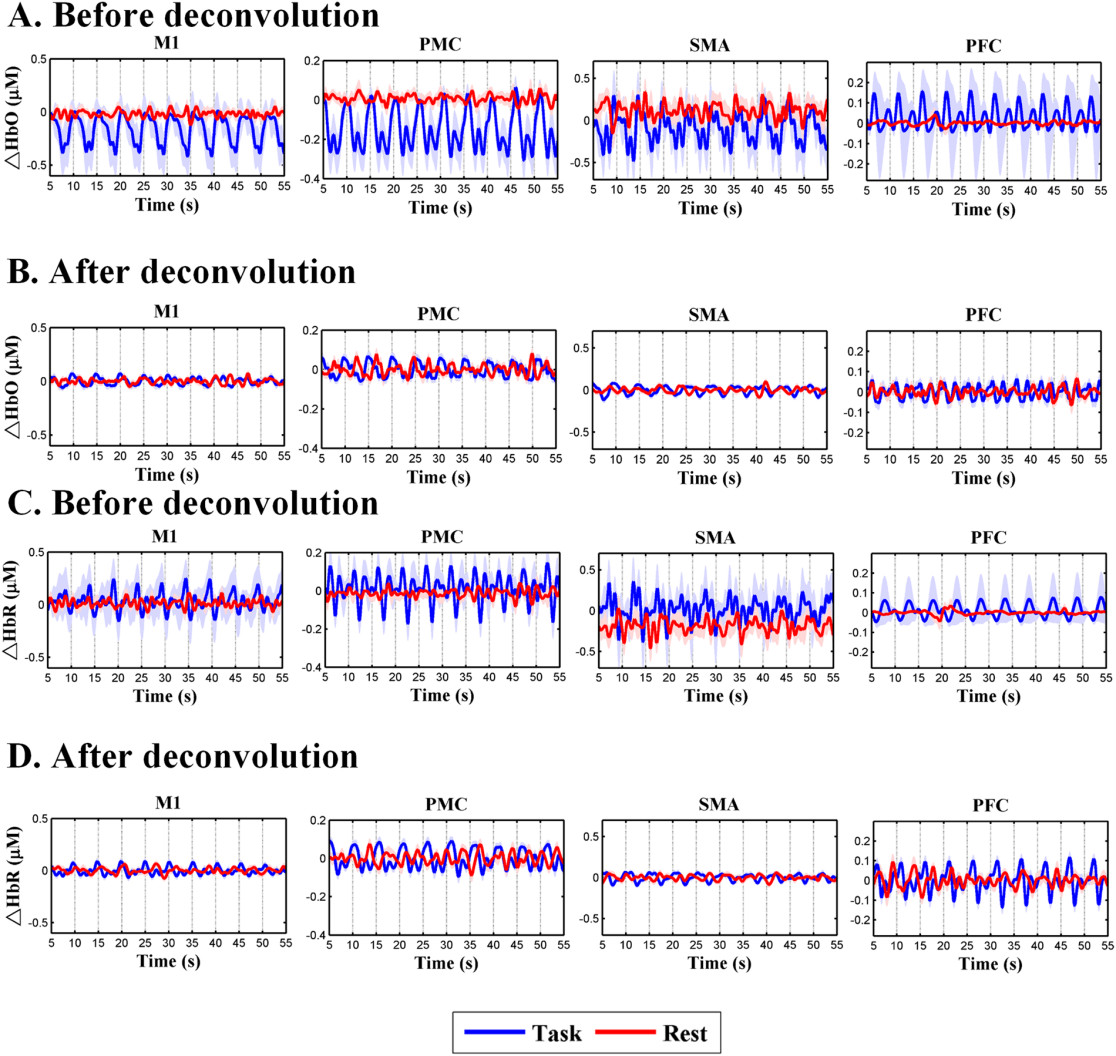
The effect of neurovascular coupling. The task-based and resting-state concentration changes in HbO (∆HbO) over the four ROIs before (**A**) and after HRF deconvolution (**B**). The task-based and resting-state concentration changes in HbR (∆HbR) from the four ROIs before (**C**) and after HRF deconvolution (**D**). The blue curves represent ∆HbR/ during performance at task-based and the red curves denote ∆HbO/∆HbR at resting-state. The pale blue lines and pale red lines represented the standard error for the task-based and resting-state recordings, respectively. M1, primary motor cortex; PMC, premotor cortex; SMA, supplementary motor area; PFC, prefrontal cortex; ROI, region of interest.

**Figure 2:**
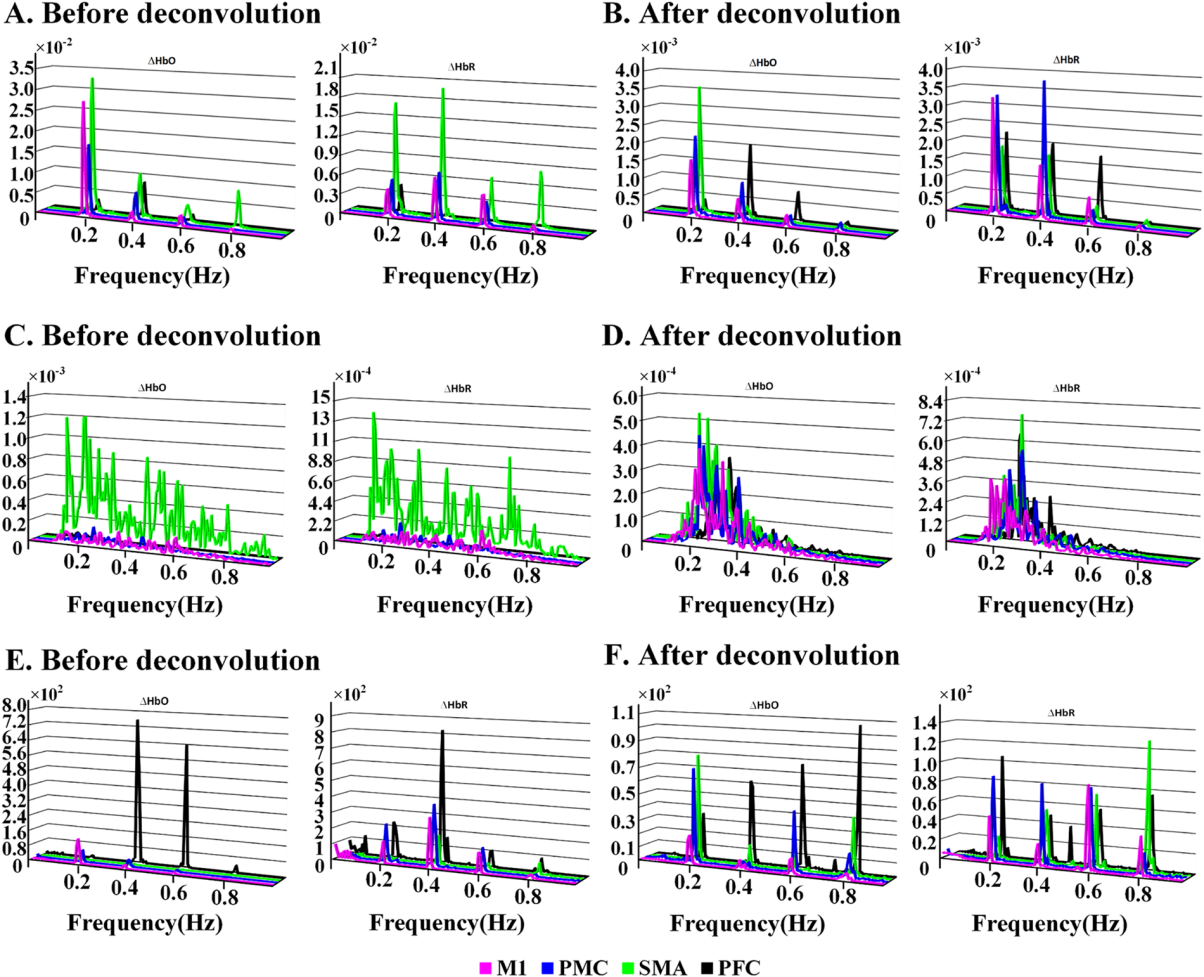
The grand average of task-evoked lfSSBR (**A,B**), and resting-state lfSSBR (**C,D**) and the relative power of lfSSBR (task divided by rest) (**E,F**) at both the fundamental frequency of stimuli (0.2 Hz) as well as its harmonics (0.4, 0.6 and 0.8 Hz). At the fundamental frequency of stimuli (0.2 Hz), the task-related lfSSBR were significantly induced.) The power of task-evoked lfSSBR before and (**A**) after HRF deconvolution (**B**). The power of resting-state lfSSBR before (**C**) and after HRF deconvolution (**D**). The relative power of lfSSBR (task/rest) before HRF (**E**) and after HRF deconvolution (**F**). lfSSBR, low frequency steady-state brain response; ∆HbO, concentration changes in oxygenated hemoglobin; ∆HbR, concentration changes in de-oxygenated hemoglobin; M1, primary motor cortex; PMC, premotor cortex; SMA, supplementary motor cortex; PFC, prefrontal cortex.

For both task-based and resting state recordings from 29 subjects, the concentration changes in HbO and HbR were calculated for each channel. Figure 1 shows the group-averaged HbO changes (∆HbO) and HbR changes (∆HbR) of each ROI before and after HRF deconvolution. It was discovered from Figs 1 and 2 that for most of the ROIs, the amplitudes of ∆HbO and ∆HbR were decreased after HRF deconvolution as compared to that before HRF deconvolution. The results indicated that the HRF deconvolution indeed changed the amplitudes of the hemodynamic responses and the power of lfSSBR^23^. However, the distribution of signal power at different frequencies didn’t exhibit significant change after HRF deconvolution, demonstrating that to some extent, the effect of lfSSBRs is independent of neurovascular coupling.

### lfSSBRs across the motor cortex at different frequencies

With periodic stimuli, we successfully evoked lfSSBR in the low frequency range underlying the SFT task. As shown in Fig. 2A, greatly increased grand averages of task-evoked lfSSBRs were identified at the frequency of 0.2 Hz and 0.4 Hz, which indicated that lfSSBRs were evoked at the fundamental frequency of stimuli (0.2 Hz) as well as its harmonics (e.g., 0.4, 0.6, and 0.8 Hz). These observations exhibited very similar distributions with that of SSEPs^37^. More importantly, task-related lfSSBRs were mostly evoked at the frequency of 0.2 Hz over the supplementary motor cortex (SMA), primary motor cortex (M1) and premotor cortex (PMC) based on HbO recordings. However, this is not the case for HbR measurements, in which task-related lfSSBRs were mostly evoked at the frequencies of 0.2 Hz and 0.4 Hz in SMA, M1 and PMC. Interestingly, before HRF deconvolution of HbO and HbR recordings, SMA exhibited group averages of resting-state lfSSBRs at the frequency bands of 0.2~0.8 Hz, whereas very weak lfSSBRs were identified in other brain regions including the M1, PMC, and prefrontal cortex (PFC) (Fig. 2C). After HRF deconvolution, participants showed high lfSSBRs across the whole motor cortex at the frequencies of 0.2 Hz and 0.4 Hz (Fig. 2D).

In addition, we also discovered from Fig. 2B,D and F that the lfSSBR waveforms were kept after the HRF deconvolution of hemodynamic signals was performed, which indicates that to some extent, lfSSBR is independent of neurovascular coupling. However, the HRF deconvolution indeed altered the distributions of power associated with lfSSBR at both the fundamental frequency of stimuli (0.2 Hz) and its harmonics (0.4, 0.6 and 0.8 Hz) although it didn’t eliminate the effect of lfSSBR.

### The correlation results

**Figure 3:**
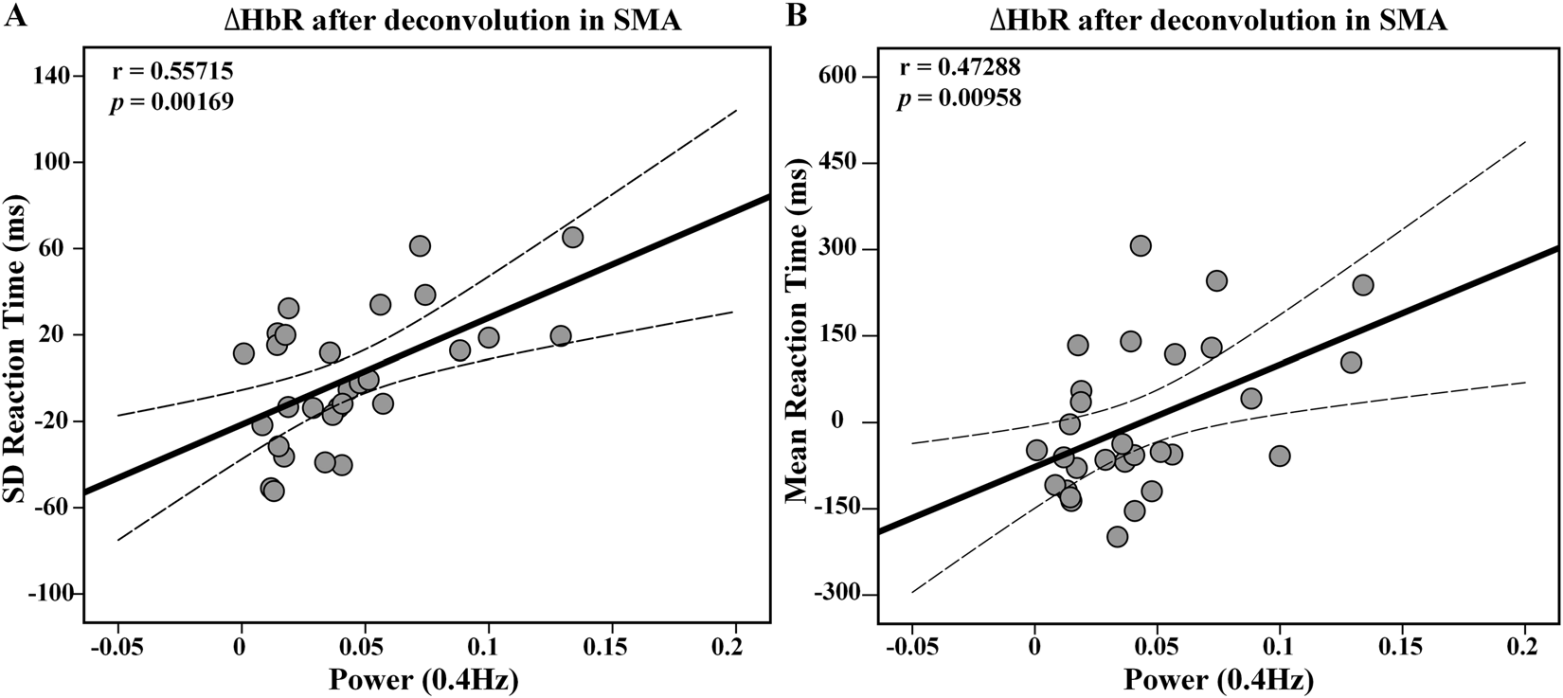
The correlation between the maximum power of task-related ∆HbR in the SMA after HRF deconvolution and the SD of reaction time (**A**), and the mean of reaction time at the frequency of 0.4 Hz (**B**). SD, standard deviation; ∆HbR, concentration changes of de-oxygenated hemoglobin; The SD of reaction time and the mean of reaction time in the figure are both residuals after age, gender and educational level regression.

After controlling for the age, gender, and educational level, the power of lfSSBR in the SMA and SD of RT showed the correlation at the frequency of 0.4 Hz after HRF deconvolution of both HbO and HbR recordings. However, a strong and significant relationship was only identified between the power of lfSSBR in the SMA and SD of RT at frequency of 0.4 Hz (r = 0.55715, Fig. 3A) after HRF deconvolution of HbR. In addition, the mean RT also showed significant correlation with the power of lfSSBR in the SMA at the frequency of 0.4 Hz after HRF deconvolution of HbR (r = 0.47288, Fig. 3B) although it failed to pass the Bonferroni correction. Further, regarding the resting-state power of lfSSBR and the task/rest power of lfSSBR, no significant correlations was identified between each of them and the RT. Overall, our results indicated that the behavior responses were associated with neural activities in the task-evoked lfSSBR, rather than the resting-state lfSSBR or task/rest lfSSBR.

### The signal-to-noise ratio

In this study, the ratio of task to rest to was adopted to quantify the signal-to-noise ratio (SNR). The SNR of ∆HbO after deconvolution was significantly decreased in the M1 at 0.2 Hz and 0.4 Hz as well as in the PMC at 0.2, 0.4 and 0.6 Hz than that before deconvolution (Table 2). In addition, the SNR of ∆HbO was also significantly lower at 0.2 and 0.4 Hz in the SMA and at 0.6 Hz in the PFC after deconvolution as compared with that before deconvolution (Table 2). By contrast, the SNR of ∆HbR after deconvolution was significantly decreased in the M1 at 0.2, 0.4, and 0.6 Hz and in the PMC at 0.4 and 0.6 Hz as compared to that before deconvolution (Table 2). Additionally, the SNR of ∆HbR after deconvolution was also significantly decreased in the SMA at 0.2 Hz (Table 2). The detailed analysis results were provided in Table 2.

**Table 2:** The group comparisons of SNR in ∆HbO and ∆HbR over different brain regions before and after deconvolution.

	SNR before deconvolution	SNR after deconvolution	*t*	*df*	*p*	Cohen’s *d*
M1 in ∆HbO						
0.2 Hz	727.81 ± 1052.36	51.05 ± 110.39	3.50	28	0.002^**^	0.92
0.4 Hz	208.68 ± 320.02	25.07 ± 27.38	3.08	28	0.005^**^	0.82
PMC in ∆HbO						
0.2 Hz	1101.99 ± 2465.95	47.69 ± 91.88	2.31	28	0.029^*^	0.61
0.4 Hz	649.80 ± 1060.95	39.74 ± 67.63	3.13	28	0.004^**^	0.83
0.6 Hz	1107.43 ± 2962.19	175.07 ± 557.24	2.05	28	0.049^*^	0.45
SMA in ∆HbO						
0.2 Hz	134.88 ± 156.97	32.68 ± 40.66	3.42	28	0.002^**^	0.91
0.4 Hz	172.81 ± 327.83	49.41 ± 72.44	2.15	28	0.040^*^	0.53
PFC in ∆HbO						
0.6 Hz	11368.34 ± 20586.20	203.96 ± 280.76	2.95	28	0.006^**^	0.78
M1 in ∆HbR						
0.2 Hz	450.74 ± 648.19	15.05 ± 9.34	3.63	28	0.001^**^	0.97
0.4 Hz	193.98 ± 276.56	42.07 ± 58.15	3.11	28	0.004^**^	0.77
0.6 Hz	822.75 ± 1464.43	109.49 ± 111.64	2.64	28	0.013^*^	0.70
PMC in ∆HbR						
0.4 Hz	931.75 ± 2380.22	22.34 ± 23.28	2.06	28	0.049^*^	0.55
0.6 Hz	384.73 ± 594.83	46.47 ± 64.68	3.15	28	0.004^**^	0.81
SMA in ∆HbR						
0.2 Hz	101.64 ± 124.11	18.29 ± 16.23	3.62	28	0.001^*^	0.96

Group comparisons: paired *t*-tests. The values are displayed as mean ± SD. SNR, signal-to-noise ratio; M1, primary motor cortex; PMC, premotor cortex; SMA, supplementary motor area; PFC, prefrontal cortex; ∆HbO, changes in concentration of oxygenated hemoglobin; ∆HbR, changes in concentration of de-oxygenated hemoglobin. ^*^Indicated significant results for *p* < 0.05, ^**^indicated significant results for *p* < 0.01.

### Optical mapping of lfSSBR in the motor system

**Figure 4:**
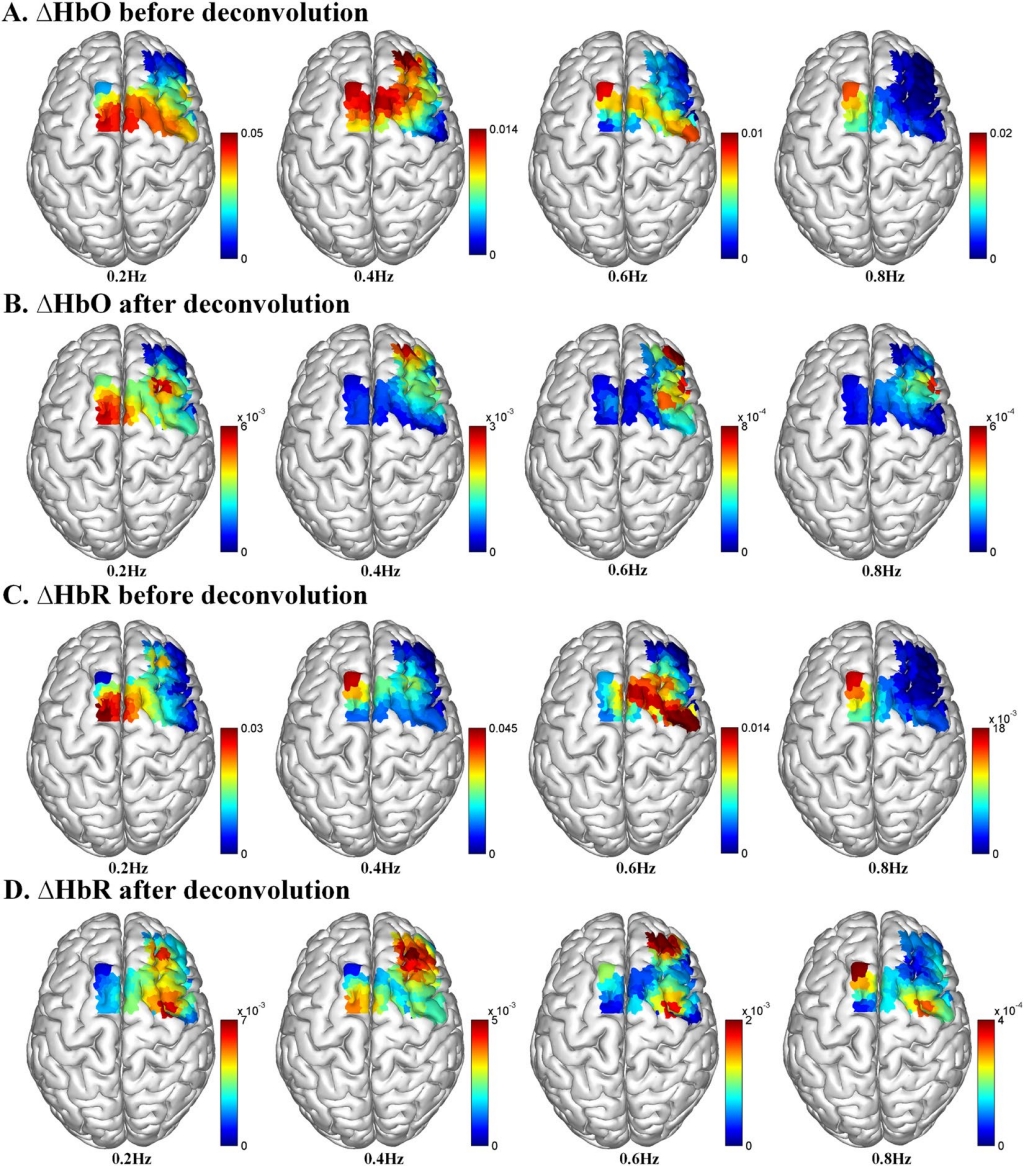
Spatial mapping of the grand-averaged task-evoked lfSSBR at the fundamental frequency of stimuli (0.2 Hz) as well as its harmonics (0.4, 0.6 and 0.8 Hz) in each channel. The power of task lfSSBR in ∆HbO before (**A**) and after HRF deconvolution (**B**). The power of task-evoked lfSSBR in ∆HbR before (**C**) and after HRF deconvolution (**D**). lfSSBR, low frequency steady-state brain response; ∆HbO, concentration changes in oxygenated hemoglobin; ∆HbR, concentration changes in de-oxygenated hemoglobin. The color bar scale denotes the lfSSBR value of each channel. The rendering was generated by using the BrainNet Viewer (http://www.nitrc.org/projects/bnv/).

Figure 4A–D shows the surface rendering of group averages of task-evoked lfSSBRs for each channel before and after HRF deconvolution of HbO and HbR signals, respectively, generated by using the BrainNet Viewer (http://www.nitrc.org/projects/bnv/). The optical mapping images were then visualized on a brain cortex template as illustrated in Fig. 4, in which lfSSBR exhibited significantly enhanced brain activity at the frequency of 0.2 Hz for the motor cortex. However, this is not the case for the prefrontal area, in which lfSSBR showed significantly enhanced brain activity at the frequency of 0.4 Hz.

### The dominant frequency of lfSSBR in the motor system

We discovered from the Fig. 2E that the highest SNR (task/rest) for both ∆HbO and ∆HbR measurements was identified at around 0.4 Hz before HRF deconvolution. We also found that the strongest correlation between the power of lfSSBR and the mean and SD of RT was identified at the frequency of 0.4 Hz in the SMA (Fig. 3). All these findings indicated that the domain frequency of lfSSBR was around 0.4 Hz in the motor system.

## Discussion

lfSSBR is a promising technique in elucidating cognition-evoked neural oscillations in the low frequency range. The present work examined the lfSSBR by using fNIRS technique which revealed different brain oscillation patterns of HbR and HbO signals. Three essential topics related to lfSSBR were inspected here: 1) Corrected the HRF independent characteristics of lfSSBR by demonstrating that lfSSBR involved both components of neural oscillations and neurovascular coupling; 2) Identified the dominant frequency of task-evoked neural oscillations during performance of the serial finger tapping task, which was at 0.4 Hz; 3) Constructed the non-linear properties of lfSSBR by task-evoked stimuli.

### The characteristics of lfSSBR evoked by finger tapping task

Our findings suggested that lfSSBRs were able to be evoked at both the fundamental frequency of stimuli (0.2 Hz) as well as its harmonics (0.4, 0.6 and 0.8 Hz), in which the mechanism of lfSSBR is very similar to that of SSEPs. More specifically, the harmonic phenomenon is widely recognized due to the non-linearly coupled neural systems. The between-frequency coupling at both the fundamental frequency of stimuli and its harmonics has been examined by EEG^20^, which exhibits the intrinsic characteristics of dynamic brain activities.

Specifically, as shown in Fig. 2, the distribution of lfSSBR exhibited the clear difference with the power distribution of brain activities at the resting state, indicating that the strength of lfSSBR, just like other brain stimulation-evoked responses (e.g., transcranial alternating-current stimulation), is determined by the natural frequency of local neural oscillations^3^ and brain states during the performance of the cognition tasks^38^. As such, the physiological meaning of lfSSBR should not only be assessed by the amplitude but also by the phase^21,24^. Although we have measured the linear relationship between lfSSBR and behavioral performance, the non-linear property of lfSSBR shows its advantages in elucidating brain functions. For instance, the physiological significance of lfSSBR could be revealed by the lfSSBR itself, without considering the resting baseline and the superposition relationship between the resting state and task state^22^ (also see The correlation results subsection). This phenomenon demonstrates there exists a non-linear relationship between the resting and task state. Recently, the negative interaction between the resting and task state has been revealed^39^, which exhibited the phase dependent^40^, supporting our hypothesis that the phase of lfSSBR carries information about psychological activities^21,24^. The phase synchronization of lfSSBR provides a good model to study the relationship between the task and resting state, which deserves further studies.

### lfSSBR incorporates both neural oscillations and neurovascular coupling components

The spatial distribution of lfSSBR is not only constrained by the natural frequency of local neural oscillations and brain states, but also influenced by the neurovascular coupling. Previous work showed that regarding lfSSBR, the neurovascular coupling can change its dominant frequency from low (<0.1 Hz) to high (>0.1 Hz) frequencies^12^. In this study, the low frequency HRF was deconvoluted, leading to more impact on the power at low frequencies than that at high frequencies. Therefore, lfSSBR with low frequencies is more influenced by the HRF deconvolution. However, the neurovascular coupling effect can also exist after HRF deconvolution, indicating that lfSSBR involves both neural oscillations and neurovascular coupling components.

Although the signal of fNIRS has been recognized to be regulated by neurovascular coupling, the present results show that lfSSBR survives after HRF deconvolution, suggesting that this index can reveal neural level activities^35^. Just like lfSSBR quantified by fMRI, the steady-state response can modulate neural activities at a particular frequencies, endowing it the power to explore cognitive-based neural oscillations^21–24^. Unlike the GLM which is heavily dependent of neurovascular coupling, the lfSSBR assesses the non-linear brain activities with extremely high SNR (ten to hundreds for lfSSBR as shown in Fig. 2F vs. 5% for brain activation)^12,23,37^. Due to the close relationship between the low frequency BOLD signal and neural oscillations^41,42^, the high SNR signal of lfSSBR may provide a sensational measurement of low frequency neural oscillations. Further, the waveform of lfSSBR is sine wave^24^ rather than that of traditional HRF. Beyond the large peak, the great trough may also be related to the balance of excited and inhibited neural firing^43^, suggesting that there exists a close relationship between the neural activity and lfSSBR. Overall, the lfSSBR may provide a different measurement to explore the non-linear neural activities at the low frequency range.

### Different mechanisms of HbO and HbR

Typically, neural activities induced local changes in cerebral blood flow, cerebral blood volume and oxygenation in the brain, a biophysical phenomenon which was called the neurovascular coupling^44,45^. Both fMRI and fNIRS take advantage of this phenomenon by measuring the hemodynamic correlations of neural activity. Different from fMRI that relies on the paramagnetic properties of BOLD contrast, fNIRS is based on the intrinsic optical absorption of blood and can measure the concentration changes of both HbO and HbR simultaneously^46,47^. In response to the brain neural activity, increases in local blood flow, blood volume and oxygenation are directed to the regions, resulting in changes in concentration of HbO and HbR, i.e., an increase in oxygenated blood and a decrease in deoxygenated blood. However, there are controversies on which hemoglobin species best represents the cortical activation and BOLD response. For example, the first fused fNIRS-fMRI study on humans conducted by Kleinschmidt *et al*. demonstrated that BOLD signals were correlated strongly with decreased concentration changes of HbR^48^. Additional work showed that BOLD signals were also related with total hemoglobin concentrations changes^49,50^. In particular, Okamoto *et al*. discovered the equal correlations between the BOLD signals and concentration changes of both HbR and HbO^51^. However, it is widely recognized that an increase in BOLD contrast and a drop in HbR correlated rather well^52^. Thus, there may not be a single best hemoglobin species to reveal the brain activations and consequently it is preferable to analyze both the HbO and the HbR signals.

Interestingly, in our study, only HbO is correlated with blood oxygenation and is the dominated component of blood volume, in which the increase in oxygenated blood is accompanied by a decrease in deoxygenated blood. Consequently, the HbO and HbR chromophores can exhibit different absorption spectra of near-infrared light (wavelengths 650~1000 nm), which further causes different concentration changes of HbO and HbR. More importantly, the changes in the concentration of these chromophores can be used as surrogate markers of the brain blood oxygenation and blood volume, thus providing a means of investigating brain functions. The different concentration changes in HbO and HbR may lead to the different power of them after FFT analysis, thus generating different patterns of lfSSBR.

### The dominant frequency of the motor system was located at 0.4 Hz

Previous lfSSBR studies showed that sensorimotor regions rather than higher cognitive related regions can survive in higher frequencies^21–23^, indicating that dominant frequency of sensorimotor regions may be relatively high. In the present study, the highest SNR (task/rest) was identified at the frequency of 0.4 Hz in both ∆HbO and ∆HbR before HRF convolution. Besides, we observed that the strongest correlation between the SMA and the mean and SD of RT was identified at 0.4 Hz, suggesting that the dominant frequency of SMA may be around this frequency. The mean and SD of RT exhibited the neural activity-related human behaviors, whereas the lfSSBR after HRF deconvolution showed the behavior-associated neural activity. The strongest correlation between the lfSSBR in the SMA and the mean and SD of RT was identified at the frequency of 0.4 Hz, which demonstrated that the dominate frequency for the neural activity should be around this frequency since the dominate frequency can induce the strongest lfSSBR. Combined together, we can conclude that the domain frequency was at the frequency of 0.4 Hz. Consequently, the dominate frequency of motor systems and associated lfSSBR are evoked by cognitive tasks such as a plan of action or motor execution action rather than external low-frequency electromagnetic stimuli. It is independent of the neurovascular coupling, which, to a large degree, can reflect neural-level low-frequency brain activities. Finally, the mechanism for the dominate frequency of sensory motor remains largely unclear. However, we discovered from our results that 0.4 Hz was more likely close to the dominate frequency of the motor system as compared with 0.2 Hz, since only dominate frequency can induce the strongest lfSSBR. Our findings also indicated the frequency-specific of the motor task. However, the exact dominant frequency band should be further investigated using other task with different stimulus frequencies, such as 0.1 Hz, 0.3 Hz, and 0.6 Hz, to eliminate the influence of HRF and harmonic effect, which is able to precisely localize the dominant frequency band.

In particular, lfSSBRs were identified to have an enlarged variability when the stimuli were operated at a constant frequency. The increased variability is essential for the neural systems to operate in an optimal way. The significant variability of brain oscillations can generate large dynamic ranges for the brain to pick up a proper response from many states^53^. As a result, the variability is crucial for the flexibility, efficiency and adaptability of the neural system. In particular, lfSSBRs are able to reveal more insightful evidences than the brain activations in studies of life-span development, skill learning, and training in terms of the adaptive hypothesis.

The band-limited method can be adopted to identify the neural basis of the lfSSBR with low-frequency neural oscillations. In addition, although the HRF deconvolution has been explored, which can generate the neural level signals in theory, simultaneous EEG and fNIRS recordings should be performed later in order to reveal the realistic neural oscillations mechanism of lfSSBR. What’s more, lfSSBR should be performed to examine multiple cognitive tasks at different frequencies in the future. Further, the physiological signals such as heart rate, blood pressure and skin blood flow are not measured in the present study and they cannot be ruled out by the HRF deconvolution. Since most of the studies on the BOLD signals cannot completely rule out these effects^54^, we cannot state that we capture the pure neural-level signals. However, in this study, to reduce the effect of physiology noise on lfSSBR to the greatest extent, the data was processed by a bandpass filter of a high cut off filter at 0.8 Hz and a low cut off filter at 0.1 Hz. For example, the blood pressure oscillation that is generally less than or around 0.1 Hz was almost eliminated from our results in Fig. 2 and Table 2. The high-frequency heart rate signal, which is generally higher than 1 Hz, was not revealed in the results in Fig. 2 as well. The breath and body movement-related noises were eliminated by principle component analysis. The only concerns is the background effect including skin blood flow, which is not related to neural activity or neurovascular coupling. However, the background noise including skin blood flow signals can be reduced by removal of the effect of resting-state recordings (by the calculation of task/rest).

## Conclusions

Our findings suggested that the lfSSBR is independent of the neurovascular coupling to some degree, which can reveal the underlying process of neural oscillations. Consequently, the lfSSBR showed its potential as an effective tool to study low-frequency oscillations that might reflect the neural-level signals. This study is, as far as we know, an account of the first ever study to investigate the lfSSBR at low-frequency bands using fNIRS. We have successfully induced the lfSSBR in the low frequency bands and demonstrated that the dominant frequency of the motor system was at the frequency of 0.4 Hz. As an essential tool in examining the neural activities, lfSSBR can provide us novel and critical insights into neuroplasticity and low-frequency neural oscillations during the completion of various cognitive tasks.

## Methods

### Participants

Twenty-nine healthy college students (14 males and 15 females, mean age ± standard deviation: 24.24 ± 1.38, ranging from 21 to 27 years) were recruited from the campus of University of Macau. The detailed information about the age, gender and educational level was provided in Table 3. All participants were right-handed, who were assessed by using the Chinese revised-version of Edinburgh Handedness Inventory^55,56^. All subjects were required to sign the informed consent documents after a full description of the study before the experimental tests. All clinical tests were approved by the Biomedical Ethics Board with Faculty of Health Sciences at the University of Macau (Macao SAR, China) and all methods were performed in accordance with the relevant guidelines and regulations. All subjects had normal or corrected-to-normal vision. Participants with reported history of neurological or psychiatric disorders were excluded from this study. Subjects who drank alcohol or caffeine or took any psychoactive medicines within 24 hours prior to the experiment were also excluded from the present work.

**Table 3:** The demographic characteristics of all the subjects.

Subject	Gender	Age (years)	Educational level (years)
1	Male	25	17
2	Male	26	17
3	Female	23	17
4	Female	26	17
5	Female	24	17
6	Male	24	17
7	Female	24	18
8	Female	24	17
9	Male	25	17
10	Male	24	17
11	Male	25	17
12	Female	25	18
13	Male	23	15
14	Male	23	16
15	Male	25	16
16	Male	24	16
17	Male	24	16
18	Male	26	16
19	Male	27	20
20	Male	22	16
21	Female	24	17
22	Female	24	16
23	Female	24	17
24	Female	24	17
25	Female	23	16
26	Female	25	18
27	Female	22	16
28	Female	27	17
29	Female	21	14

### Tasks and Procedures

**Figure 5:**
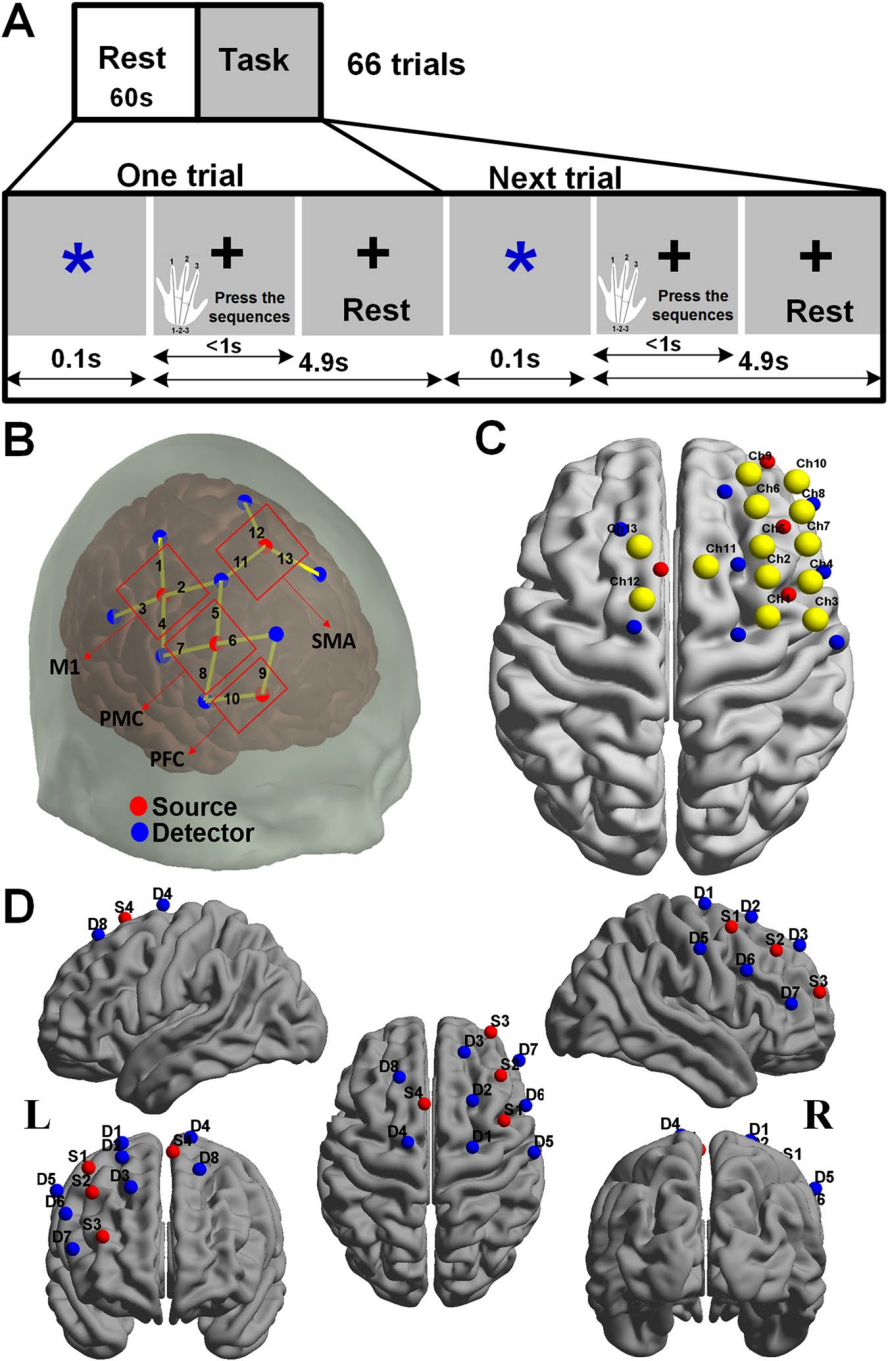
The task design for fNIRS and probe arrangements and 3D channel locations for the subjects. (**A**) The session includes 66 trials. Each trial consists of a 0.1 s blue asterisk and a 4.9 s fixation. Subjects were required to press three fingers orderly as fast and accurately as possible when they saw the asterisk. The reaction time was determined by the interval between the onset of the presentation of the asterisk and the complete of the key-press response. (**B**) The red and blue solid circles indicate the positions of emitters and detectors, respectively. The red rectangles represent the ROIs defined in this study including the M1, the PMC, the SMA, and the PFC and the numbers stand for the emitter-detector pairs, i.e. channels. (**C**) Co-registered positions of channels on a standard brain atlas. The yellow circles denote the 13 channels. (**D**) Co-registered configurations of the optodes on a standard brain atlas. The brain image was visualized with the BrainNet Viewer (http://www.nitrc.org/projects/bnv). The anatomical position of each channel on the brain atlas is reported in detail in Table 4. M1, primary motor cortex; PMC, premotor cortex; SMA, supplementary motor area; PFC, prefrontal cortex; ROI, region-of-interest; L, left hemisphere; R, right hemisphere.

The fNIRS data was acquired in two sections: one for resting state and the other for SFT task. The resting state recordings lasted 10 minutes, during which all participants were instructed to move as little as possible, keep their eyes closed, and think nothing in particular during scanning period. For the second section, subjects were required to perform a SFT task, as shown in Fig. 5A. The frequency of the SFT task was set at 0.2 Hz and consequently the duration for each trial was 5 seconds. During the stimuli period, a blue asterisk (stimulus) was first presented at the center of the screen. The blue asterisk remained on the screen for 0.1 s and participants were asked to press the sequences as fast and accurately as possible. Then a post-stimulus and recovery period for 4.9 s with a black fixation cross was displayed in the center of the monitor. The order of finger movement sequences was 1, 2 and 3, in which “1” corresponded to the ring finger, “2” corresponded to the middle finger, and “3” corresponded to the index finger. Subjects placed their index finger, middle finger, and ring finger of left hand on the horizontally placed keyboard in the numeric keypad. Altogether, the fNIRS recordings for the second session consisted of 66 sequences (trials) and the data acquisition lasted about 330 seconds. During the performance of the SFT task, the subjects were instructed to remain completely focused without counting or thinking about the stimuli.

**Table 4:** The corresponding MNI coordinate, AAL, and Brodmann area for each channel.

Channels	Estimated MNI			BA	AAL	Probability
	x	y	z			
Ch1	36	−2	66	6	R SFG	0.99
Ch2	36	14	63	8	R MFG	0.55
Ch3	54	−3	55	6	R PreCG	0.87
Ch4	52	12	49	6	R MFG	0.46
Ch5	34	26	56	8	R MFG	0.58
Ch6	32	42	47	9	R MFG	0.99
Ch7	51	27	41	44	R MFG	0.51
Ch8	49	40	31	45	R MFG	0.78
Ch9	29	55	36	46	R MFG	0.49
Ch10	47	52	18	46	R MFG	0.79
Ch11	13	18	69	6	R SMA	0.62
Ch12	−11	5	74	6	L SMA	1
Ch13	−12	26	65	8	L SMA	0.86

BA, Brodmann’s area; AAL, anatomical automatic labeling; MNI, Montreal Neurological Institude; Ch, channel; R, right; L, left; SFG, superior frontal gyrus; MFG, middle frontal gyrus; PreCG, precentral gyrus; SMA, supplementary motor area.

### Data Acquisition

The experiments were performed using a continuous wave (CW) fNIRS system (Techen, Inc., Milford, MA, USA). In our system, two CW lights at wavelengths of 690 nm and 830 nm are emitted at each source fiber, which is able to provide sensitive detection for the changes of both HbO and HbR concentrations in the human brain. The distance between each source and detector pair was set to 30 mm and the sampling rate for the present study was 50 Hz. The present configurations of the source and detector pairs were shown in Fig. 5B–D, in which a total of 4 light source emitters and 8 detectors were connected by the optical fibers on the scalp to generate 13 channels.

The middle central (Cz) position of the international 10–20 system was the marker for locating the motor and frontal areas. In particular, we placed the probe based on some reference points including the Nasion, Inion, Cz, the left and right preauricular points. A three dimensional (3D)-magnetic space digitizer (Polhemus Inc.) was used to measure the 3D spatial location of each fNIRS channel and each optode. The averaged 3D coordinates were further imported to NIRS-SPM software for spatial registration to generate the distribution of the optodes and channels, and the Montreal Neurological Institute (MNI) coordinates of the channels^57^. Then the optodes and channels in the brain were visualized by using the BrainNet Viewer^58^, as showed in Fig. 5C and D. Interestingly, it was discovered from Fig. 5B–D that the right M1 was covered by channels 1–4, PMC was covered by channels 5–8, SMA was covered by channels 11–13, and PFC was covered by channels 9 and 10.

### Signal Preprocessing

The preprocessing of fNIRS signals was performed using the HomER2 software (http://homer-fnirs.org/)^59^. The motion artifacts of the acquired fNIRS raw data were detected and removed first. Then the raw data was processed by a bandpass filter of a low cut off filter at 0.1 Hz and a high cut off filter at 0.8 Hz in order to eliminate effect of physiological and instrumental noise^60–62^. The high cut filter can remove the high-frequency measurement noise while the low cut filters can remove the slow physiological noise such as blood pressure oscillations. In addition, the filtered optical density (OD) signals were converted to the hemoglobin concentration changes at different time points according to the modified Beer-Lambert law^63,64^. Further, trials with incorrect responses were discarded for further analysis. Finally, the averaged HbO/HbR was calculated for each channel, and the grand-averaged hemodynamic responses from all 29 subjects were also generated and shown in Fig. 1.

### The HRF Deconvolution and Power Analysis

To examine whether the lfSSBR was really independent of neurovascular coupling, the HRF deconvolution operation was performed to generate the “neural level signals” (lfSSBR after HRF deconvolution). HbO and HbR signals were first used and the onsets of neural events were kept for the HRF recovery. The HRF in each channel was generated by matching HbO and HbR measurements with the canonical HRF and its time derivative. The HRF was based on the convolution of the boxcar function and the sum of two gamma functions as the canonical HRF which has been mentioned in Uga’s study^65^. And then signals at the neural level were reconstructed by using Winener deconvolution (http://users.ugent.be/)^35^.

**Figure 6:**
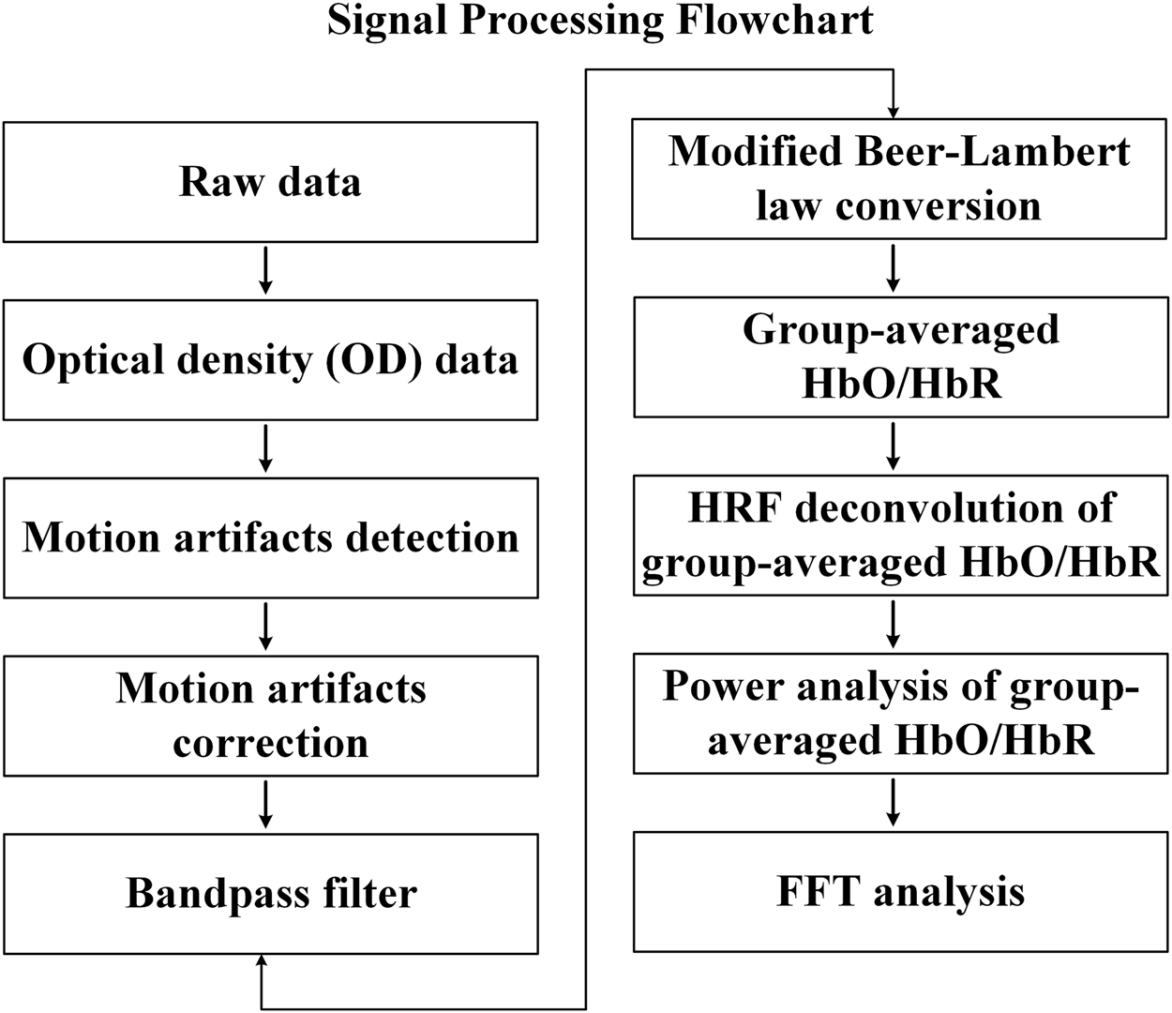
The processing flowchart for fNIRS raw data in the current study.

The power analysis is able to provide the power distribution of a signal across all the frequencies. In this study, the ∆HbO and ∆HbR time series from each ROI (M1, PMC, SMA, and PFC) after group average were converted to the frequency-domain signals using a Fast Fourier Transform (FFT) algorithm. These time series were also detrended to remove the baseline shifts before applying the FFT. The flowchart for the operation procedure was provided in Fig. 6. The FFT was the most widely used method to define the SSEPs in EEG studies, which was adopted here as well to generate the distributions of lfSSBRs in frequency domain. The power from all ROIs before and after HRF deconvolution was calculated underlying the task and resting-state conditions. Meanwhile, the Pearson’s correlation analysis was performed between the power of ∆HbO or ∆HbR at frequencies of 0.2 Hz, 0.4 Hz, 0.6 Hz and 0.8 Hz from all the ROIs and the RT of each subject before and after HRF deconvolution, controlling for the age, gender, and educational level.
